# Elevated Serum D-Dimer May Reflect the Presence of Gut Inflammation in Spondyloarthritis

**DOI:** 10.3389/fmed.2021.816422

**Published:** 2022-01-21

**Authors:** Jiaqi Feng, Jia Li, Yixuan Li, Yuyang Jin, Fang Du, Xiaoxiang Chen

**Affiliations:** Department of Rheumatology, School of Medicine, Renji Hospital, Shanghai Jiao Tong University, Shanghai, China

**Keywords:** spondyloarthritis, d-dimer, gut inflammation, peripheral joint involvement, ileo-colonoscopy

## Abstract

**Background:**

To investigate the association of D-dimer with gut inflammation in spondyloarthritis (SpA).

**Methods:**

Sixty-five patients with SpA and 70 healthy controls were included. Demographic, clinical, and laboratory parameters were collected. The differences of clinical and laboratory parameters were compared between patients with SpA and healthy controls, and between patients with SpA, with and without gut inflammation. The associations of D-dimer with laboratory data were analyzed. The predictive value of D-dimer was obtained by a receiver operator characteristic (ROC) curve analysis. The independent risk factors for gut inflammation in SpA were investigated by binary logistic regression analysis.

**Results:**

Patients with SpA had higher D-dimer than healthy controls *(P* = 0.016). D-dimer was positively correlated with platelet (PLT), erythrocyte sedimentation rate (ESR), and C-reactive protein (CRP), and negatively correlated with hemoglobin (Hb). Besides, significant differences were observed in D-dimer between SpA patients with and without gut inflammation (*P* < 0.001). Furthermore, SpA patients with gut inflammation were more likely to have peripheral joint involvement than those without gut inflammation (*P* < 0.001). The AUC of D-dimer was 0.865 at cut-off value of 0.29 mg/L, with a sensitivity of 82.6%, and a specificity of 81%. Elevated D-dimer (OR = 15.451, 95% CI: 3.030–78.780, *P* = 0.001) was independently associated with gut inflammation in SpA.

**Conclusion:**

D-dimer may be a potential biomarker for identifying SpA patients with gut inflammation.

## Introduction

Spondyloarthritis (SpA) is a common chronic inflammatory disease, affecting the 0.2–1.61% of the population (1, 2). The SpA is currently classified as axial spondyloarthritis [axSpA, including ankylosing spondylitis (AS)] and peripheral spondyloarthritis (pSpA). The axSpA mainly affects spine or sacroiliac joints, with or without peripheral joint involvement (PJI), while pSpA refers to the condition where only the peripheral joint is involved (arthritis, enthesitis, or dactylitis) (3, 4). In addition, extra-articular manifestations are common, including anterior uveitis (AU), psoriasis, and inflammatory bowel disease (IBD), including Crohn's disease (CD), and ulcerative colitis (UC) (5).

Gut inflammation includes both clinical and subclinical gut inflammation. While the former is well known to be in the form of CD, or UC, the latter tends to be asymptomatic (6). The prevalence of clinical gut inflammation (including IBD) is in the range of 6 to 14% among patients with SpA, while the incidence of subclinical gut inflammation is up to 44-60% (7). On the other hand, SpA occurs frequently in patients with IBD, ranging from 3.7 to 13% (8, 9). Moreover, patients with SpA have an increased risk of developing IBD (10), and the gut inflammation is related to a higher disease activity of axSpA (11). Currently, accumulated evidence indicates that there exists a great amount of genetic, immunological, and pathophysiologic overlap between the SpA and gut inflammation (12, 13).

Ileo-colonoscopy and biopsy, which are regarded as gold standard tools, are currently required for the confirmation of gut inflammation (14). In fact, gut inflammation often occurs asymptomatically, and patients are unwilling to undergo the ileo-colonoscopy for its discomfort, invasiveness, and expense, with a resultant missed diagnosis and the delayed treatment. Therefore, an effective screening tool is warranted for identifying patients with SpA who are likely to have a suspected gut inflammation.

D-dimer serves as a biomarker of both fibrin formation and degradation, reflecting the activated coagulation and fibrinolysis. Besides the thrombotic disorders, cancers, and infection, D-dimer is increased in autoimmune disease (15, 16). Vascular injury and vascular thrombosis are found to be involved in IBD, resulting in an increase in D-dimer (17, 18). Moreover, the D-dimer is elevated in axSpA and is associated with the disease activity (19, 20). The current study is to investigate the association of D-dimer with gut inflammation in SpA.

## Materials and Methods

### Patients

A total of 65 patients in the Department of Rheumatology, Shanghai Renji Hospital, from July 2019 to July 2020, and 70 healthy controls (HC) were recruited in this study. Each patient was diagnosed with SpA, fulfilling the classification criteria of the Assessment of Spondyloarthritis International Society criteria (ASAS) (3, 4). The ASAS was used to classify the patients as affected by axial and/or peripheral SpA. All patients have no previous history of infectious diseases, diabetes, hematological diseases, arterial, or venous thrombosis. All patients have given their informed consent prior to their inclusion in the study. This study was performed in accordance with the Declaration of Helsinki and with the ethical standards of the institutional research committee. Ethical approval was obtained by Renji Hospital Ethics Committee, Shanghai Jiaotong University School of Medicine (reference number: 2017-201).

### Data Collection

Clinical and laboratory data were collected. Clinical manifestations included sacroiliitis, PJI, gastrointestinal (GI) symptoms, anterior uveitis, psoriasis, and gut inflammation. PJI was defined as the presence of peripheral arthritis, enthesitis, or dactylitis. Laboratory data involved whole blood cell counts, erythrocyte sedimentation rate (ESR), C-reactive protein (CRP), and human leukocyte antigen B27 (HLA-B27). The coagulation parameters include D-dimer, fibrinogen (FIB), prothrombin time (PT), activated partial thromboplastin time (APTT), and thrombin time (TT).

### Ileo-Colonoscopic and Histologic Examination

Ileo-colonoscopy was performed among 65 patients with SpA, permitting visualization of ileum, colon, and rectum. Meanwhile, biopsy was performed when macroscopic change was found over the surface of the digestive duct. The obtained mucosal tissues were embedded in 40% paraffin, cut in 3 mm sample sections, and stained with H&E for histological evaluation. The primary diagnosis of gut inflammation was made both macroscopically and microscopically according to mucosal lesion and inflammatory cell infiltration, as well as with the previous reports (21, 22).

### Statistical Analysis

Data were analyzed using IBM SPSS Statistics 25 software and GraphPad Prism 8 software. Quantitative data were expressed as median and interquartile range (IQR), and categorical variables were presented as numbers and percentages. The Mann-Whitney U test or Student's *t*-test was used for continuous variables, and the chi-square test or Fisher's exact test was used for categorical variables. Spearman's correlation analysis was used to assess the associations of D-dimer with other laboratory parameters. The receiver operator characteristic (ROC) curve was used to discriminate between patients who had gut inflammation from those who did not. We determined the cut-off value and calculated the areas under the curve (AUC), sensitivity, and specificity to compare the applicability of the variables. All variables with *P* < 0.1 on univariate analysis were selected for the multivariate logistic regression analysis [forward: likelihood ratio (LR)] to explore the factors that are independently associated with gut inflammation in patients with SpA. Results were presented as odds ratios (OR) with 95% CI. All tests were two-tailed. The value of *P* < 0.05 were considered statistically significant.

## Results

### Demographic, Laboratory, and Clinical Characteristics Between Patients With SpA and Healthy Control

Among the 65 patients with SpA, 56 were diagnosed with axSpA (22 with PJI) and nine were diagnosed with pSpA. Thirty-two patients had PJI, 23 patients had gut inflammation, four patients had anterior uveitis, and no patient had psoriasis. Demographic, laboratory, and clinical characteristics of patients with SpA and healthy controls were listed in Table 1. D-dimer was significantly higher in patients with SpA than healthy controls (*P* = 0.016). Moreover, significant differences between patients with SpA and HC were observed in terms of PLT, Hb, ESR, CRP, and FIB (*P* < 0.05, respectively). There were no significant differences between the two groups in terms of PT, APTT, and TT (*P* > 0.05, respectively).

**Table 1:** Demographic, laboratory, and clinical characteristics of the subjects.

	**Healthy control**	**SpA patients**	***P* value**
	**(*n* = 70)**	**(*n* = 65)**	
Age, yrs	33.5 (28.8–37.3)	29.0 (25.0–39.0)	0.288
Sex, male/female	59/11	61/4	0.102
PLT,10^∧^9/L	232 (191–249)	253 (219–310)	**0.043**
Hb, g/L	148(139–156)	142(125–149)	**0.010**
ESR, mm/h	5(2–9)	21(7–53)	**<0.001**
CRP, mg/L	0.4 (0.2–4.3)	7.5 (7.2–37.2)	**<0.001**
D-dimer, mg/L	0.14 (0.11–0.32)	0.21 (0.13–0.73)	**0.016**
FIB, g/L	2.23 (1.83–2.60)	3.10 (2.31–4.19)	**<0.001**
PT, s	11.6 (11.2–12.3)	11.7 (11.2–12.4)	0.628
APTT, s	30.5 (29.4–32.3)	31.0 (29.8–33.6)	0.169
TT, s	17.3 (16.9–17.7)	17.7 (17.0–18.6)	0.100
axSpA/pSpA		56/9	
HLA-B27 positivity, n (%)		58 (89.2)	
Sacroiliitis, n (%)		56 (86.2)	
Peripheral joint involvement, n (%)		31(47.7)	
Peripheral arthritis		29	
Enthesitis		5	
Dactylitis		6	
GI symptoms, n (%)		9 (13.8)	
Anterior uveitis, n (%)		4 (6.2)	
Psoriasis, n (%)		0(0)	
Gut inflammation, n (%)		23 (35.4)	

*PLT, platelet; Hb, hemoglobin; ESR, erythrocyte sedimentation rate; CRP, C-reactive protein; FIB, fibrinogens; PT, prothrombin time; APTT, activated partial thromboplastin time; TT, thrombin time; HLA-B27, Human Leukocyte Antigen B27; GI, gastrointestinal. Bolded values indicate P < 0.05*.

### The Association of D-Dimer With Other Laboratory Parameters

**Figure 1:**
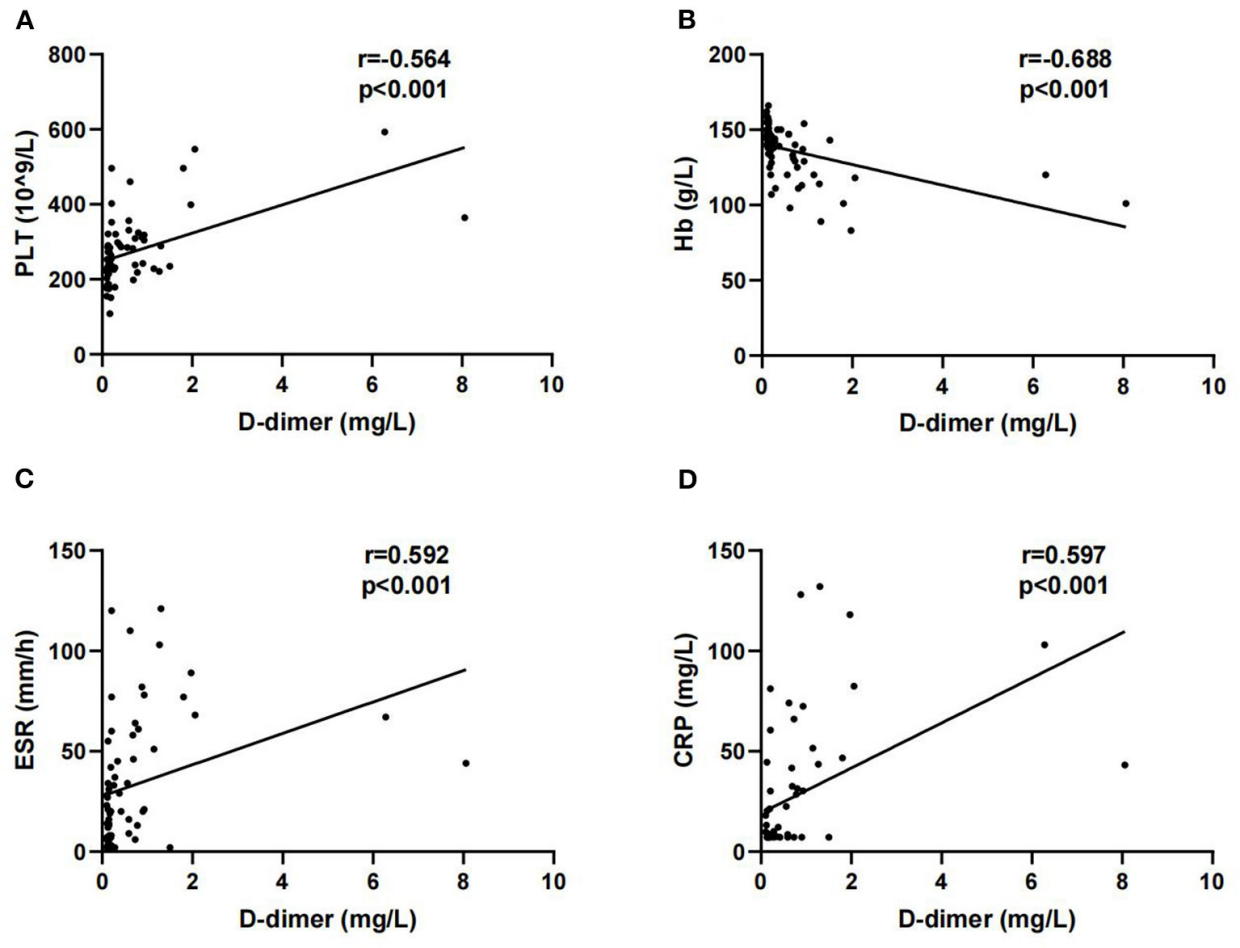
Correlations of D-dimer with platelet (PLT) **(A)**, Hb **(B)**, erythrocyte sedimentation rate (ESR) **(C)** and C-reactive protein (CRP) **(D)**.

Next, we analyzed the correlations between D-dimer and other laboratory parameters in patients with SpA. D-dimer was positively correlated with PLT (r = 0.564, *P* < 0.001), ESR (r = 0.592, *P* < 0.001), and CRP (r = 0.597, *P* < 0.001). In addition, a negative correlation was observed between D-dimer and Hb (r = −0.688, *P* < 0.001) (Figure 1).

### The Relationship Between D-Dimer and Clinical Features

**Figure 2:**
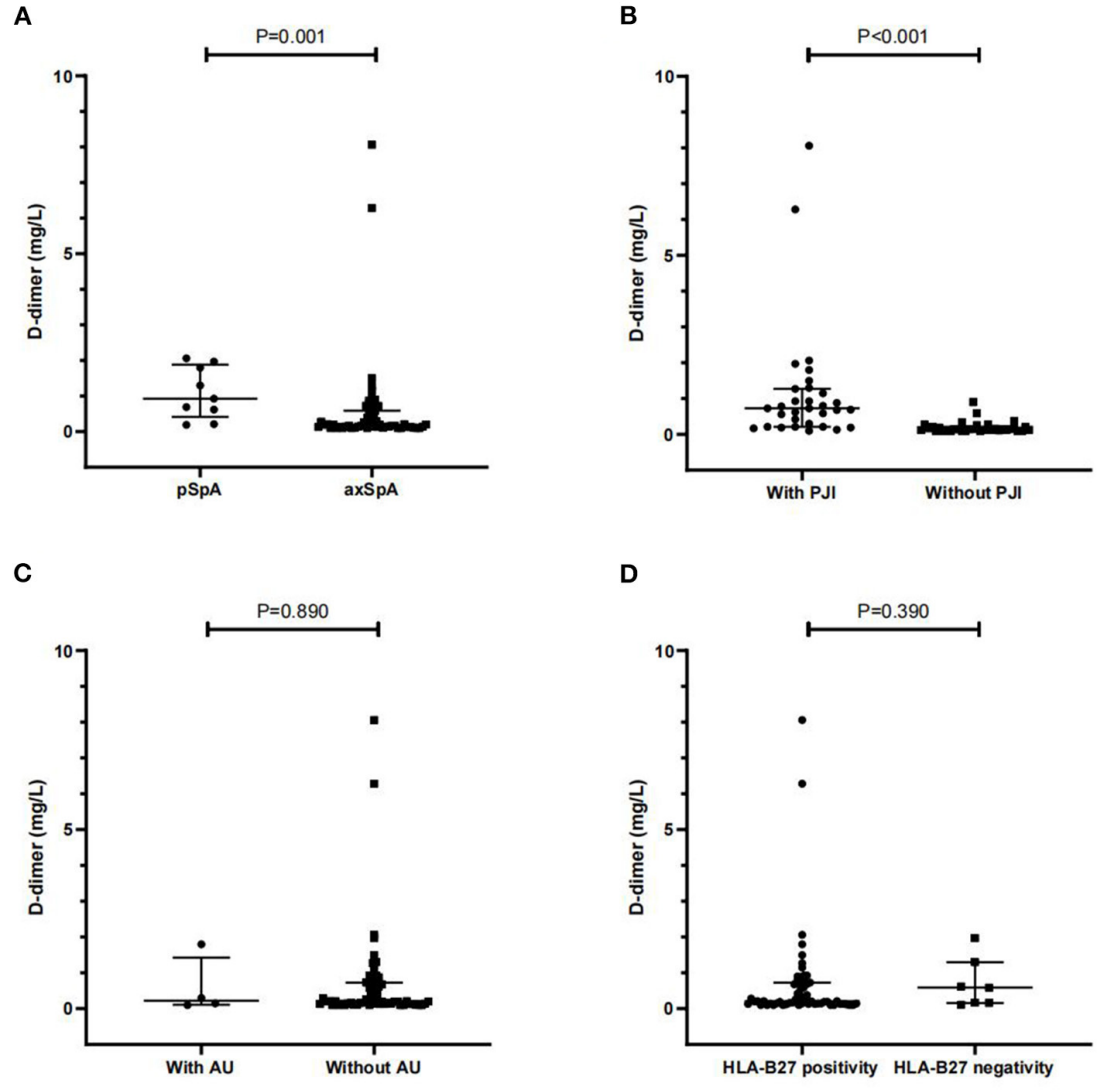
Comparisons of D-dimer between axial spondyloarthritis (axSpA) and pSpA patients **(A)**, and between SpA patients with and without PJI **(B)**, AU **(C)** and HLA-B27 positivity **(D)**. *PJI* peripheral joint involvement; *AU* anterior uveitis; HLA-B27 human leukocyte antigen B27. Data are expressed as median and interquartile range (IQR).

We further analyzed the relationship between D-dimer and the clinical features. Overall, patients with pSpA had a significantly higher level of serum D-dimer than patients with axSpA [0.93 (0.42–1.89) mg/L vs. 0.19 (0.13–0.59) mg/L, *P* = 0.001]. Additionally, patients with SpA with PJI had a significantly higher D-dimer than those without PJI [0.73 (0.21–1.27) mg/L vs. 0.15 (0.11–0.20) mg/L, *P* < 0.001]. In addition, no significant difference was observed in D-dimer between patients with SpA, both with and without AU, and between patients with SpA with HLA-B27 positivity, and those with HLA-B27 negativity (*P* > 0.05, respectively) (Figure 2).

### Ileo-Colonoscopic and Histopathological Findings

**Figure 3:**
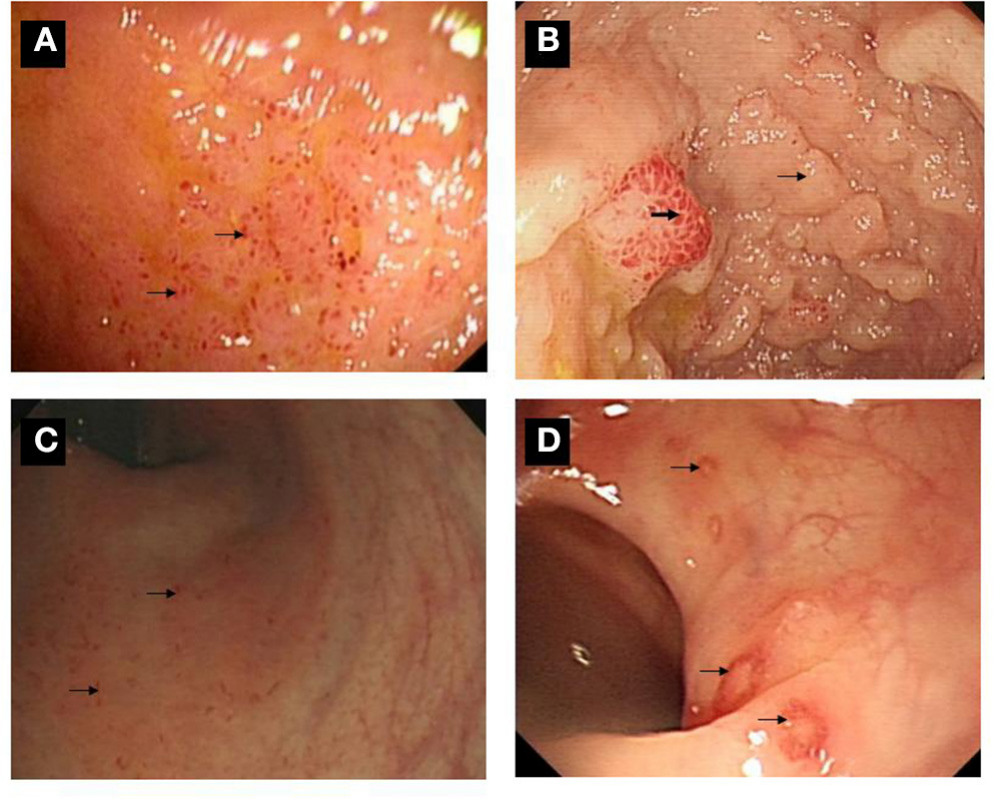
Ileo-colonoscopic pictures of gut inflammation among SpA patients. **(A)** Multiple lesions were over the surface in the terminal ileum. **(B)** A sign of mucosal nodularity (small arrow) with hyperemia-like changes was in terminal ileum (bold arrow). **(C)** Multiple small lesions were found over the surface of rectum and sigmoid colon (arrow). **(D)** Multiple ulcers were found over the surface of descending, sigmoid colon and rectum (arrow).

**Figure 4:**
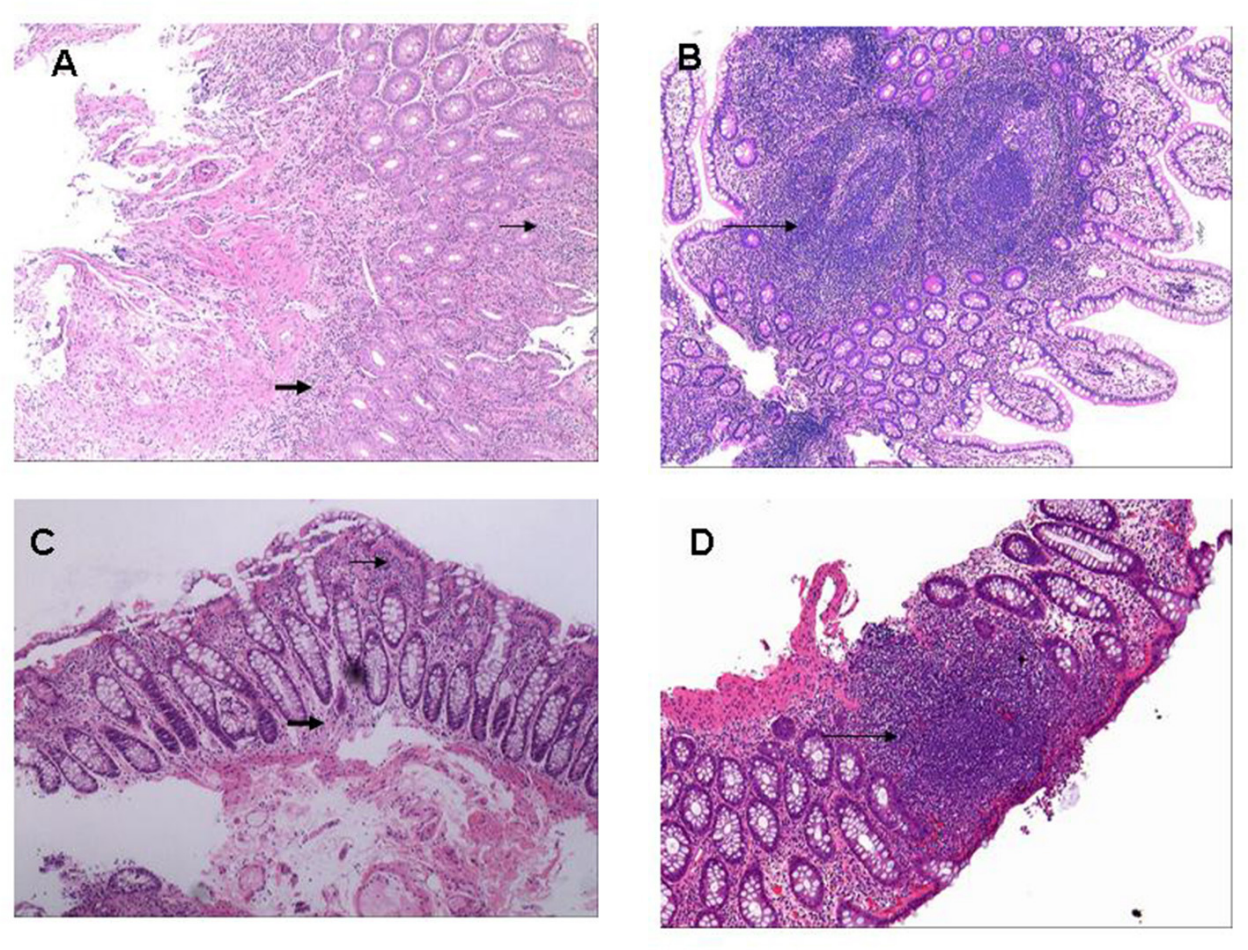
Histopathological pictures of gut inflammation among SpA patients. **(A)** Histologically infiltration of Inflammatory cells was around gland duct (small arrow) and at underlying submucosa (bold arrow) located in terminal ileum (H&E; original magnification ×10). **(B)** Multiple ectopic lymphoid tissues were microscopically observed in the terminal ileum (long arrow) with massive infiltration of inflammatory cells around gland duct (H&E; original magnification ×10). **(C)** Tissue specimen from descending colon demonstrated moderate infiltration of inflammatory cells around gland duct (small arrow) and in the underlying submucosa (bold arrow) (H&E; original magnification ×20). **(D)** A ectopic lymphoid tissue was microscopically observed at rectum specimen (long arrow) with massive infiltration of inflammatory cells around gland duct (H&E; original magnification ×20).

Overall, 23 patients had macroscopic lesion over the surface of digestive duct, ranging from erythema, oedema, friability of the mucosa, mucosal erosion to ulcerations (Figure 3). Macroscopic lesion was detected in the terminal ileum in 12 cases, in the colon of nine patients, and in both ileum and colon of two patients were affected. Histologically, inflammatory cell infiltration was found around the gland or in the underlying submucosa in all cases, four cases had abundant neutrophil infiltration, and 10 cases had lymphocyte aggregation or lymphoid tissue formation (Figure 4). Four (6.2%) patients were diagnosed with UC and 3 (4.6%) with CD.

### The Comparisons of Clinical and Laboratory Data Between Patients With and Without Gut Inflammation

The clinical and laboratory characteristics of patients with and without gut inflammation were listed in Table 2. There were 35.4% (23/65) patients with SpA with gut inflammation. Among these patients, gut inflammation was detected in 32.1% (18/56) patients with axSpA and 55.6% (5/9) in patients with pSpA, with no significant difference between them. However, significant difference was observed in terms of the prevalence of PJI between patients with and without gut inflammation (*P* < 0.001). There was no significant difference in terms of the prevalence of AU and HLA-B27 positivity between the two groups. In general, patients with SpA with gut inflammation had higher disease activity than those without gut inflammation, including PLT, ESR, and CRP (*P* < 0.05, respectively). Of note, among the coagulation parameters, D-dimer level was significantly higher in patients with gut inflammation than those without gut inflammation (*P* < 0.001), while there was no significant difference in terms of FIB, PT, APTT, and TT between them (*P* > 0.05, respectively). Moreover, Hb concentration was significantly lower in patients with gut inflammation (*P* = 0.002).

**Table 2:** Comparisons of demographic, laboratory, and clinical characteristics of patients with SpA between with and without gut inflammation.

	**Gut inflammation (–) (*n* = 42)**	**Gut inflammation (+) (*n* = 23)**	***P* value**
Age, yrs	29.0 (25.0–35.3)	32.0 (25.0–43.0)	0.219
Sex, male/female	40/2	21/2	0.610
PLT,10^∧^9/L	233 (198–286)	293 (235–331)	**0.008**
Hb, g/L	145 (134–150)	129 (114–140)	**0.002**
ESR, mm/h	18 (5–39)	37 (13–67)	**0.045**
CRP, mg/L	7.2 (7.0–18.6)	28.6 (7.2–66.1)	**0.006**
D-dimer, mg/L	0.15 (0.12–0.23)	0.73 (0.38–1.30)	**<0.001**
FIB, g/L	2.94 (2.27–4.13)	3.35 (2.97–4.34)	0.308
PT, s	11.7 (11.2–12.2)	11.8 (11.2–12.8)	0.206
APTT, s	30.9 (29.8–33.5)	31.0 (29.8–33.6)	0.678
TT, s	17.9 (16.9–18.7)	17.5 (17.1–18.4)	0.553
axSpA/pSpA	38/4	18/5	0.260
HLA-B27 positivity, n (%)	38 (90.5)	20 (87.0)	0.691
Sacroiliitis, n (%)	38 (90.5)	18 (65.2)	0.260
Peripheral joint involvement, n (%)	13 (31.0)	18 (78.2)	**<0.001**
Peripheral arthritis	12	17	
Enthesitis	3	2	
Dactylitis	3	3	
Anterior uveitis, n (%)	3 (7.1)	1 (4.3)	1.000
GI symptoms, n (%)	3 (7.1)	6 (26.1)	0.057
Loose stools	2	3	
Abdominal pain	1	3	

*PLT, platelet; Hb hemoglobin; ESR, erythrocyte sedimentation rate; CRP, C-reactive protein; FIB, fibrinogens; PT, prothrombin time; APTT activated partial thromboplastin time; TT, thrombin time; HLA-B27, Human Leukocyte Antigen B27; GI, gastrointestinal. Bolded values indicate P < 0.05*.

### ROC Analysis of Laboratory Parameters for Detecting the Gut Inflammation in SpA

**Figure 5:**
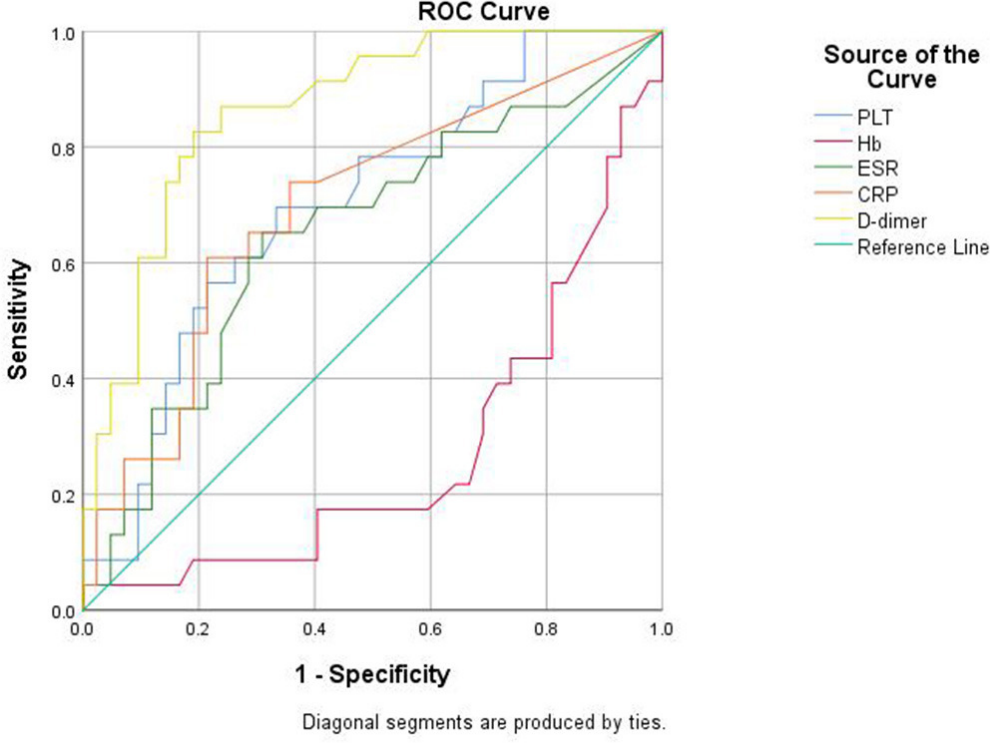
Receiver operator characteristic (ROC) curve analysis of the biomarkers for detecting the gut inflammation in patients with SpA.

We further explored the predicting efficiency of these parameters (including PLT, Hb, ESR, CRP, and D-dimer) for gut inflammation in patients with SpA. The ROC analysis was shown in Table 3 and Figure 5. Among these parameters, the AUC of D-dimer was 0.865 (95% CI: 0.777–0.954; *P* < 0.001), greater than other parameters. The optimum cut-off point of D-dimer was 0.29 mg/L, accompanied by a sensitivity of 82.6% and a specificity of 81%.

**Table 3:** Predictive values of the biomarkers for detecting the gut inflammation of patients with SpA.

	**AUC**	**Cutoff point**	**Sensitivity**	**Specificity**	***P* value**	**95%CI**
PLT (10^∧^9/L)	0.700	260	69.6%	66.7%	**0.008**	0.569–0.831
Hb (g/L)	0.271	141	21.7%	66.7%	**0.002**	0.140–0.402
ESR (mm/h)	0.651	28.5	65.2%	69.0%	**0.046**	0.507-0.794
CRP (mg/L)	0.697	20.8	60.9%	78.6%	**0.009**	0.560–0.833
D-dimer (mg/L)	0.865	0.29	82.6%	81.0%	**<0.001**	0.777–0.954

*PLT, platelet; Hb, hemoglobin; ESR, erythrocyte sedimentation rate; CRP, C-reactive protein; AUC, areas under the ROC curve; 95% CI, 95% confidence interval. Bolded values indicate P < 0.05*.

### Binary Logistic Regression Analysis of Gut Inflammation

Binary logistic regression analysis was performed to investigate the factors that are independently associated with gut inflammation in SpA. The results indicated that an increased D-dimer (OR = 15.451, 95% CI: 3.030–78.780, *P* = 0.001) was independently associated with gut inflammation in SpA (Table 4).

**Table 4:** Binary logistic regression analysis of gut inflammation in SpA.

**Risk factors**	**Univariate analysis**		**Multivariate analysis**	
	**OR (95%CI)**	***P* value**	**OR (95%CI)**	***P* value**
Age	1.039 (0.989–1.092)	0.128	-	-
Male	0.525 (0.069–3.997)	0.534	-	-
PLT	1.006 (1.000–1.013)	**0.034**	-	-
Hb	0.956 (0.929–0.989)	**0.008**	-	-
ESR	1.015 (0.999–1.032)	**0.068**	-	-
CRP	1.019 (1.002–1.037)	**0.030**	-	-
D-dimer	15.451 (3.030–78.780)	**0.001**	15.451 (3.030–78.780)	**0.001**
FIB	1.133 (0.800–1.605)	0.481	-	-
PT	1.668 (0.843–3.298)	0.142	-	-
APTT	1.073 (0.931–1.237)	0.331	-	-
TT	0.781 (0.480–1.271)	0.320	-	-
pSpA	2.639 (0.632–11.020)	0.183	-	-
HLA-B27 positivity	0.702 (0.143–3.448)	0.663	-	-
Sacroiliitis	0.379 (0.091–1.583)	0.183	-	-
Peripheral joint involvement	8.031 (2.450–26.326)	**0.001**	-	-
Anterior uveitis	0.591 (0.058–6.029)	0.657	-	-
GI symptoms	4.588 (1.025–20.530)	**0.046**	-	-

*PLT, platelet; Hb hemoglobin; ESR, erythrocyte sedimentation rate; CRP, C-reactive protein; FIB, fibrinogens; PT, prothrombin time; APTT, activated partial thromboplastin time; TT, thrombin time; pSpA, peripheral spondyloarthritis; HLA-B27, Human Leukocyte Antigen B27; GI, gastrointestinal; OR, odds ratio; 95% CI, 95% confidence interval. Bolded values indicate P < 0.05*.

## Discussion

In the present study, our results demonstrated that D-dimer was correlated with inflammatory index. Moreover, D-dimer was increased in patients with SpA with PJI, as well as in patients with SpA with gut inflammation, and an elevated D-dimer was independently associated with gut inflammation in SpA. Additionally, patients with gut inflammation were more likely to have PJI than those without gut inflammation.

The D-dimer is a biomarker that reflects fibrin formation and degradation, while FIB is the precursor to fibrin, which is produced in the liver. The increased D-dimer has been reported in conditions with thrombosis (16), IBD (17, 23), rheumatoid arthritis (RA) (24, 25), and SpA (20), while FIB increases under injury, infection, or inflammation. It is reported that the activation of coagulation and fibrinolysis system was also shown in either intra-articular synovial tissues of arthritis (20) or intestinal tissues of IBD (18). Other reports suggested that the vascular injury or vasculitis might be involved in the pathogenesis of IBD (26, 27). The prevailing opinion is that coagulation system is activated excessively in the setting of inflammation. Furthermore, the mutual interaction between coagulation and inflammation may amplify the activation of both systems and may accelerate the development of disease (28). The present study demonstrated that D-dimer in patients with SpA was increased compared with healthy controls and was correlated with blood parameters (PLT, ESR, and CRP), and was consistent with the report that the coagulation system was activated in axSpA (29). Other studies also indicated that D-dimer was increased and was paralleled with the disease activity in RA (25, 30). Additionally, patients with SLE in the active group had significantly higher D-dimer level than those in the inactive and control groups (31). Also, D-dimer was significantly increased in patients with adult-onset Still's disease (AOSD) with macrophage activation syndrome (MAS), compared with those without MAS (32). In addition, we also found that the Hb concentration is negative with D-dimer level.

As for the relationship between D-dimer and different clinical features, we found that D-dimer level was significantly increased in patients with pSpA than with axSpA. Furthermore, we demonstrated that patients with PJI have higher level of D-dimer than those without PJI. In fact, the relationships between D-dimer and PJI among patients with SpA have not been reported. Nevertheless, this association can also be found in RA and in patients with juvenile idiopathic arthritis (JIA), which are both characterized by peripheral joint inflammation (25, 33).

Regarding the association of D-dimer with gut inflammation among patients with SpA, we demonstrated that these patients with SpA with gut inflammation had a higher disease activity including an increased ESR and CRP than those without gut inflammation. We also found that patients with SpA with gut inflammation had both an increased D-dimer and decreased Hb, resulted from the possible involvement of vascular injury and small vessel thrombosis in the pathogenesis (18, 26, 27). Similarly, others have shown that D-dimer level was increased in active IBD group, compared with patients with inactive IBD and healthy controls, with average of 0.326 vs. 0.229 mg/L and 0.243 vs. 0.140 mg/L, respectively (17, 34). In our study, ROC analysis demonstrated that the cut-off value of 0.29 mg/L was obtained at optimal AUC of 0.865 for identifying gut inflammation in SpA, with a sensitivity of 82.6% and a specificity of 81%.

Concerning the screening tool, few biomarkers and predictive model have been studied. Fecal calprotectin (FC) serves as a potential biomarker for small bowel inflammation, with an AUC of 0.75 (FC > 132 μg/g), accompanied by a sensitivity of 66.7%, and a specificity of 76.9% (35). Furthermore, FC could be used to detect a suspected gut inflammation and to identify patients with AS at an increased risk of developing IBD (at a baseline threshold of 266 mg/kg). Currently, the cut-off value of FC is reported to be of great variability (36–38). Also, MIRJAM D suggested that perinuclear anti-neutrophil cytoplasmic antibody (pANCA) might be a predictive indicator to perform endoscopy (39). At present, there is no simple and convenient biomarker for predicting the gut inflammation in SpA. On the other hand, a multiparametric model [including age, male gender, Bath ankylosing spondylitis disease activity index (BASDAI), and Bath ankylosing spondylitis metroloty index (BASMI)] was established for predicting microscopic gut inflammation in axSpA, with a sensitivity of 81.8% and a specificity of 78.3% (11). In our study, we did not compare the predicting efficiency between D-dimer and FC, pANCA or the aforementioned model, but the serum D-dimer test should be a simple and stable tool although it remains to be validated in a larger scale. Moreover, logistic regression analysis in our study indicated that an elevated D-dimer is independently associated with the gut inflammation in SpA. To our knowledge, this is the first description of this association of an elevated D-dimer with gut inflammation among the population with SpA.

Interestingly, our results primarily showed that PJI is associated with gut inflammation. The current classification criteria for SpA are based on the location of the involved joints instead of its pathophysiology. According to the criteria, axSpA includes the involvement of axial skeleton with or without peripheral joint inflammation. However, the criteria now began to gain debates (3, 40, 41), and the focus is whether axSpA and pSpA share a same pathogenic origin. Currently, it is generally accepted that both have overlapping biological backgrounds: genetic factors play a more closely related role in axial involvement than peripheral joint inflammation, while the peripheral joint involvement is reported to be linked with gut inflammation (42–44). Furthermore, recent advances suggested that there existed differences between them concerning immunological alterations and response to biologic disease-modifying anti-rheumatic drugs (bDMARDs) (13). Therefore, for the clinical purpose, it is important to identify potential patients with SpA with gut inflammation. We also found that it is not the axial involvement, but the PJI that is related to gut inflammation, similar to the previous findings that the patients with axSpA with subclinical gut inflammation (as assessed by FC levels) is closely linked with peripheral joint inflammation (45). Nevertheless, we did not observe that there was a significant difference in terms of the prevalence of gut inflammation between axSpA and pSpA. These results indicated that gut inflammation is more frequently seen in SpA with PJI regardless of the SpA classification. In addition, an elevated D-dimer was not only observed in patients with SpA with PJI, but also in patients with SpA with gut inflammation, suggesting there is a possible link between PJI and gut inflammation. Above all, our results suggested that PJI might be a distinct phenotype that is associated with gut inflammation.

This study has several limitations. First, our sample size is relatively small. Second, the conventional ileo-colonoscopy, instead of the capsule endoscopy (CE), was performed. The CE may detect suspected small bowel inflammation in patients with SpA with elevated D-dimer, permitting greater visualization. Third, we did not evaluate the gut inflammation, failing to explore the association between D-dimer and the disease activity of gut inflammation. Above all, larger multicenter studies should be conducted for further validation.

In conclusion, our study indicates that the elevated D-dimer is related to PJI and gut inflammation, and PJI is possibly associated with gut inflammation. Furthermore, we suggest, for the first time, that serum D-dimer may be a potential biomarker for identifying patients with SpA with suspected gut inflammation, but the determination of cutoff value is required in a larger study.

## Data Availability Statement

The raw data supporting the conclusions of this article will be made available by the authors, without undue reservation.

## Ethics Statement

The studies involving human participants were reviewed and approved by the Ethics Committee of Renji Hospital, Shanghai, China. The patients/participants provided their written informed consent to participate in this study.

## Author Contributions

JF and JL designed the research studies, acquired data, analyzed data, and wrote the manuscript. YL and YJ acquired data. FD and XC checked the medical charts. XC designed the research studies, revised the article, and provided the mentorship of this study. All the authors contributed to the article and approved the submitted version.

## Funding

This study was supported by the National Natural Science Foundation of China (Grants 81771729, 81971534).

## Conflict of Interest

The authors declare that the research was conducted in the absence of any commercial or financial relationships that could be construed as a potential conflict of interest.

## Publisher's Note

All claims expressed in this article are solely those of the authors and do not necessarily represent those of their affiliated organizations, or those of the publisher, the editors and the reviewers. Any product that may be evaluated in this article, or claim that may be made by its manufacturer, is not guaranteed or endorsed by the publisher.
